# Supplementary material for: Glucocorticoids of European Bison in Relation to Their Status: Age, Dominance, Social Centrality and Leadership
Source: Animals (Basel). 2022 Mar 28;12(7):849. doi: 10.3390/ani12070849 (PMC8996974; doi:10.3390/ani12070849)

Supplementary File S1

Protocol to measure faecal glucocortocoids metabolites (FGM)

This laboratory protocol was performed by Rupert Palme team:
https://www.vetmeduni.ac.at/Stress-CD/EIA-protocol.pdf

Measurement with EIA

a. Buffers and solutions

We used the following buffers and solutions:

- Coating buffer: 1.59 g Na_2CO_3 (Merck 106392 or Sigma S-7795); 2.93 g $NaHCO_3$ (Merck 106329 or Sigma S-6014) dissolved and filled up to 1 l with DDW, adjust to pH 9.6 with (about 10 ml) HCl (1 mol/l)

- HCl (1 mol/l): 920 ml DDW + 80 ml 37 % HCl (Merck 100317 or Sigma H-1758)

- Assay buffer: 2.42 g Trishydroxyaminomethane (Merck 108382 or Sigma T-1503), 20 mmol/l; 17.9 g NaCl (Merck 106404 or Sigma S-9625), 0.3 mol/l) 1 g Bovine serum albumin (Sigma A-4503); 1 ml Tween 80 (Merck 822187 or Sigma P-8074) dissolved and filled up to 1 l with DDW and adjusted to pH 7.5 with (about 17 ml) HCl (1 mol/l) filter through Sep-Pak® C18 (see 1.3.1.)

- Filtration of buffer through Sep-Pak® C18: Sep-Pak® classic C18 cartridge (360 mg; Waters WAT051910) rinsed with 5 ml methanol (Merck 106009), followed by 10 ml DDW (done by hand with a syringe), connect column to tubing of peristaltic pump (flow rate of 2 to 10 ml/min), we discarded the first 10 ml of the filtrated buffer and collected buffer in clean bottle.

- "Second" coating buffer: 3.146 g Trishydroxyaminomethane (see 1.3.); 23.3 g NaCl (Merck 106404 or Sigma S-9625) 13 g BSA (Sigma A-4503); 1.3 g Sodium azide (Merck 106688) dissolved and filled up to 1.3 l with DDW and adjusted to pH 7.5 with (about 40 ml) HCl (1 mol/l), filtered trough SEP-PAK C18.

- Washing solution: 0.5 ml Tween 20 (Merck 822184); added with 2.5 l DDW

- Substrate buffer for peroxidase: 1.36 g Sodium acetate (Merck 6267) = 10 mmol/l, dissolved and filled up to 1 l with DDW and adjusted to pH 5.0 with (~8 ml) 5 % citric acid (Merck 100244)

- Enzyme solution for Streptavidin-reaction: 30 ml assay buffer + 0.001 ml Streptavidin-POD-conjugate (=0.5 U; Roche 11 089 153 001, 500 U) mixed on a magnetic stirrer a few minutes before use

- Substrate solution for peroxidase: 30 ml of substrate buffer + 0.5 ml 3,3',5,5'-Tetramethylbenzedine (0.4 %) stored in a dark bottle; (0.4 % = 0.4 g [Fluka 87748] in 100 g Dimethylsulfoxide [Fluka 41641]) + 0.1 ml H_2O_2 (0.6 %; 0.3 ml H_2O_2 [35 %, Merck 108600] + 17.5 ml DDW) mixed gently on a magnetic stirrer a few minutes before use

- Stop reagent: 2 mol/l H_2SO_4; 900 ml DDW + 100 ml H_2SO_4 (95-97 %; Merck 100731)

b. Coating of microtitre plates (with anti-rabbit IgG or Protein A)

For 30 MPT, we prepared a solution of 1 mg IgG (Sigma R2004-5x1MG; add 1 ml DDW to a portion of 1 mg) dissolved in 750 ml coating buffer (see 1.1.). We dispensed 0.25 ml/well of diluted IgG to the MTP (F96 MaxiSorp, No. 442404, Co. Nunc, Denmark). We incubated the plate at room temperature overnight. We discarded the solution and refilled each well with 0.3 ml "second" coating buffer. We covered the filled MTP with parafilm and dust cover (Nunc 264623), and kepts it at room temperature until use. We used the MTP after 3 hours.

c. Reagents

We kept all stock solutions frozen at -20°C until use. We diluted 0.01 ml of a stock solution (1 mg steroid per ml methanol) with 20 ml of assay buffer. We mixed for 2 minutes in the supersonic bath, waited 3 hours and filled portions of 0.05 ml into new vials. One vial contains 25,000 pg of the respective steroid.

d. Working dilutions

We dispensed 0.15 ml (0.2 ml when lyophilized) of assay buffer to one portion of standard vial, shake and wait 20 min. Dilute this solution 1 : 2.5 seven times. We mixed well after each step (500 pg till 2 pg per 10 µl) with a Hamilton Microlab dispenser 1000 (0.09 ml standard + 0.135 ml assay buffer).

e. Assay procedure

We washed coated MTP (see 2.) three times with washing solution. We removed the rest of liquid by blotting the MTP on paper towels. We dispensed assay buffer for nonspecific binding (NSB) and zero binding (0), standards (4.1.), pool X and Y (PX, PY), and sampled into the MTP (see Fig. below) prepared earlier (5.1.). We used the Hamilton Microlab dispenser 1000. It takes 0.01 ml from each sample and adds 0.04 ml assay buffer to dispense a total of 0.05 ml.

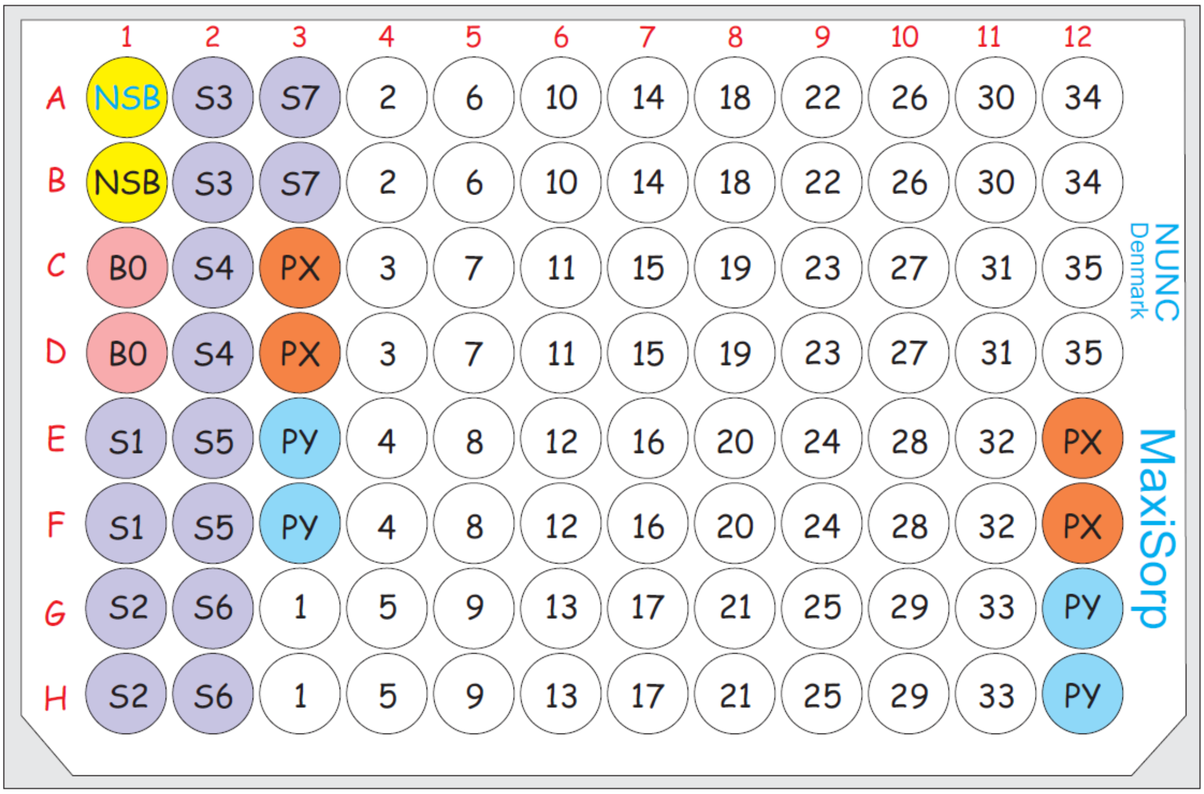

Example of arrangement of standards, pools and samples on the MTP

We dispensed 0.1 ml biotin-labelled steroid into each well. For all pipetting steps, we used a multi-pipette. We dispensed 0.1 ml antibody solution (5β-androstane-3α-o1-11,17-dione-17-CMO:BSA) into each well. We covered the MTP with parafilm and dust cover (Nunc 264623), shake (mild) the MTP overnight at 4°C. We decanted incubated MTP, and washed the MTP four times with cold (4°C) washing solution (1.5). We dispensed 0.25 ml of enzyme solution (see 1.7.) into each well and incubated the covered plate for 45 minutes at 4°C on a MTP-shaker. We performed a second wash. We dispensed 0.25 ml of substrate solution into each well and incubated in the dark the covered plate 45 minutes at 4°C (agitating). We dispensed 0.05 ml of stop reagent to stop the reaction.

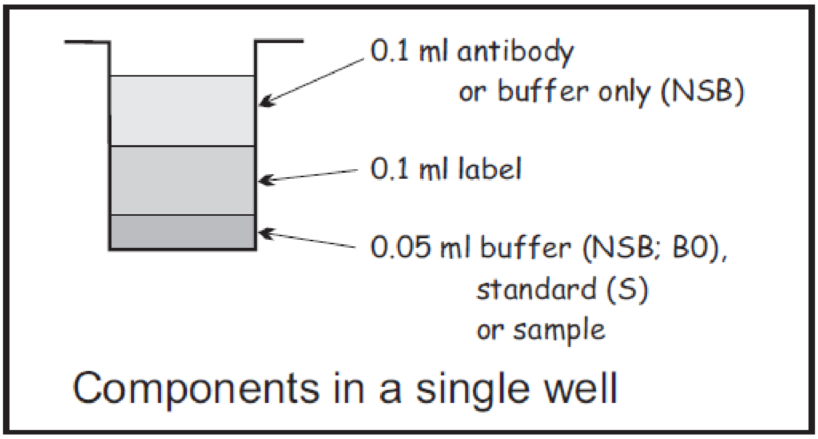

f. Absorbance measuring and calculations

We used an (automatic) MTP reader (in connection with a PC, equipped with a special software for calculation). We used an ELx808 (Bio-Tek) and Gen 5TM) (Reference filter: 620 nm; measuring filter: 450 nm).

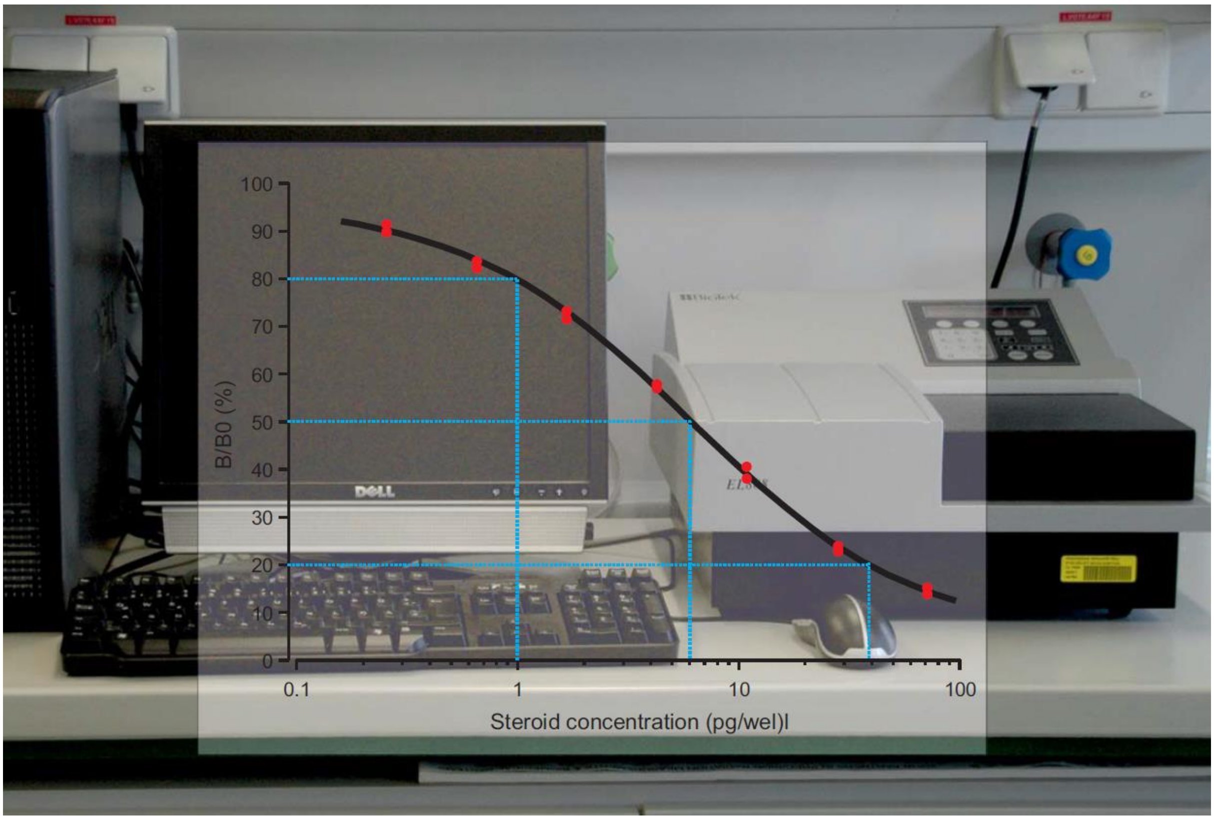

Supplement: Supplementary file 1 [file animals-12-00849-s001.zip › animals-1617984-supplementary.pdf]
